# Supplementary material for: The kinase ZYG-1 phosphorylates the cartwheel protein SAS-5 to drive centriole assembly in C. elegans
Source: EMBO Rep. 2024 May 14;25(6):2698–721. doi: 10.1038/s44319-024-00157-y (PMC11169420; doi:10.1038/s44319-024-00157-y)
Supplement: Supplementary file 2 — Dataset EV2 [file 44319_2024_157_MOESM2_ESM.docx]

| **Dataset EV2 : *C. elegans* strains and genotypes** | | | |
| --- | --- | --- | --- |
| **Strain name** | | | **Genotype** |
| N2 | | wild type var Bristol | |
| OC1268 | | *sas-5(bs308[S68A])V* | |
| OC1269 | | *sas-5(bs309[S71A])V* | |
| OC920 | | *sas-5(bs159[T101A]) V* | |
| OC923 | | *sas-5(bs160[S99A]) V* | |
| OC945 | | *sas-5(bs167[T105/S106A]) V* | |
| OC957 | | *sas-5(bs179[S99/T101A]) V* | |
| OC1000 | | *sas-5(bs201[S301/S304A]) V* | |
| OC1008 | | *sas-5(bs204[S99/T101/T105/S106A]) V* | |
| OC1030 | | *sas-5(bs211[S99/T101/T105/S106/S301/S304A) V* | |
| OC1056 | | *sas-5(bs227[S331/S338/S340A])/ nT1[qIs51] IV;V* | |
| OC1090 | | *sas-5(bs250[S10A])/nT1[qIs51] IV;V* | |
| OC1091 | | *sas-5(bs251[S10E])/nT1[qIs51] IV;V* | |
| OD1187 | | *ltSi357[pY131: sas-5p::sas-5 reencoded, cb-unc-119(+)] II; unc-119(ed3) III* | |
| OC1096 | | *ltSi357[pY131: sas-5p::sas-5 reencoded cb-unc-119(+)] II; sas-5(bs251) V* | |
| OC1097 | | *ltSi357[pY131: sas-5p::sas-5 reencoded cb-unc-119(+)] II; sas-5(bs227) V* | |
| OC1098 | | *ltSi357[pY131: sas-5p::sas-5 reencoded cb-unc-119(+)] II; sas-5(bs250) V* | |
| OC1099 | | *ltSi357[pY131: sas-5p::sas-5 reencoded cb-unc-119(+)] II; ltSi560 [pPLG014: mex-5p::GFP::his-11::tbb-2_3’utr gfp::tbg-1::tbb-2_3’utr cb-unc-119(+)] IV; sas-5(bs250) V* | |
| OC1100 | | *ltSi357[pY131: sas-5p::sas-5 reencoded cb-unc-119(+)] II; ltSi560 [pPLG014: mex-5p::GFP::his-11::tbb-2_3’utr GFP::tbg-1::tbb-2_3’utr cb-unc-119(+)] IV; sas-5(bs227) V* | |
| OC1101 | | *ltSi357[pY131: sas-5p::sas-5 reencoded cb-unc-119(+)] II; ltSi560 [pPLG014: mex-5p::GFP::his-11::tbb-2_3’-utr GFP::tbg-1::tbb-2_3’utr cb-unc-119(+)] IV; sas-5(bs251) V* | |
| OC1112 | | *ltSi357[pY131: sas-5p::sas-5 reencoded cb-unc-119(+)] II; sas-4(bs195 [sas-4::gfp] III* | |
| OC1117 | | *bsSi19[pKO120: sas-6p::superfoldergfp::sas-6_3’-utr cb-unc-119(+)] II; sas-5(bs267) V/nT1 [qIs51] IV;V* | |
| OC768 | | *bsSi28[pCW10: unc-119(+) pcdk-11.1::sfgfp::his-58::cdk-11.1 3' utr] II; unc-119(ed3) III* | |
| OC1214 | | *ltSi357[pY131: sas-5p::sas-5 reencoded cb-unc-119(+)] II; sas-4(bs195 [sas-4::gfp]) III/hT2[qIs48] I;III* | |
| OC1215 | | *ltSi357[pY131: sas-5p::sas-5 reencoded cb-unc-119(+)] II; sas-4(bs195 [sas-4::gfp]) III/hT2[qIs48] I;III; sas-5(bs250) V* | |
| OC1216 | | *ltSi357[pY131: sas-5p::sas-5 reencoded cb-unc-119(+)] II; sas-4(bs195 [sas-4::gfp]) III/hT2[qIs48] I;III; sas-5(bs251) V* | |
| OC1218 | | *ltSi357[pY131 sas-5p::sas-5 reencoded cb-unc-119(+)] II bsSi55[pNP107: cb-unc-119(+) tbb-1p::mCherry::tbb-2::tbb-2_3’-utr] IV; sas-5(bs250) V* | |
| OC1235 | | *ltSi357[pY131: sas-5p::sas-5 reencoded cb-unc-119(+)] II; bsSi55[pNP107: cb-unc-119(+) tbb-1p::mCherry::tbb-2::tbb-2_3’-utr] IV; sas-5(bs251) V* | |
| OC1236 | | *ltSi357[pY131: sas-5p::sas-5 reencoded cb-unc-119(+)] II; bsSi55[pNP107: cb-unc-119(+) tbb-1p::mCherry::tbb-2::tbb-2_3’-utr] IV* | |
| OC1237 | | *ltSi357[pY131; Psas-5::SAS-5 reencoded; cb-unc-119(+)]II; unc-119(ed3)III?; sas-6(bs188[spot::sas-6]) IV, sas-5(bs227)V* | |
| OC1238 | | *ltSi357[pY131: sas-5p::sas-5 reencoded cb-unc-119(+)] II; sas-6(bs188 [spot::sas-6]) IV* | |
| OC1239 | | *ltSi357[pY131: sas-5p::sas-5 reencoded cb-unc-119(+)] II; sas-4(bs195 [sas-4::gfp]) III/hT2[qIs48] I;III; bsSi55[pNP107: cb-unc-119(+) tbb-1p::mCherry::tbb-2::tbb-2_3’-utr] IV* | |
| OC1240 | | *ltSi357[pY131: sas-5p::sas-5 reencoded cb-unc-119(+)] II; sas-4(bs195 [sas-4::gfp]) III/hT2[qIs48] I;III; bsSi55[pNP107: cb-unc-119(+) tbb-1p::mCherry::tbb-2::tbb-2_3’-utr] IV; sas-5(bs250) V* | |
| OC1241 | | *ltSi357[pY131: sas-5p::sas-5 reencoded cb-unc-119(+)] II; sas-4(bs195[sas-4::gfp]) III/hT2[qIs48] I;III; bsSi55[pNP107: cb-unc-119(+) tbb-1p::mCherry::tbb-2::tbb-2_3’-utr] IV; sas-5(bs251) V* | |
| EG6699 | | *ttTi5605 II; unc-119(ed3) III; oxEx1578* | |
| OD1187 | | *unc-119(ed3)III; ltSi357[pY131; Psas-5::SAS-5 reencoded; cb-unc-119(+)]II* | |
| OD1702 | | *unc-119(ed3)III; ltSi560 [pPLG014; Pmex-5::GFP::his-11::tbb-2_3’UTR, GFP::tbg-1::tbb-2_3’UTR; cb-unc-119(+)]V* | |
| OD2511 | | *ltSi797[pOD1991/pSW311; Psas-5::SAS-5(S10A, S68A, S71A; reencoded); cb-unc-119(+)]II; unc-119(ed3)III* | |
| OD2512 | | *ltSi798[pOD1992/pSW312; Psas-5::SAS-5(T89A, T95A, S99A, T101A; reencoded); cb-unc-119(+)]II; unc-119(ed3)III* | |
| OD2513 | | *ltSi799[pOD1993/pSW313; Psas-5::SAS-5(T105A, S106A, S109A; reencoded); cb-unc-119(+)]II; unc-119(ed3)III* | |
| OD2514 | | *ltSi800[pOD1994/pSW314; Psas-5::SAS-5(T179A, S239A, S264A, T388A; reencoded); cb-unc-119(+)]II; unc-119(ed3)III* | |
| OD2515 | | *ltSi801[pOD1995/pSW315; Psas-5::SAS-5(T288A, S292A, S301A, S304A; reencoded); cb-unc-119(+)]II; unc-119(ed3)III* | |
| OD2516 | | *ltSi802[pOD1996/pSW316; Psas-5::SAS-5(S331A, S338A, S340A; reencoded); cb-unc-119(+)]II; unc-119(ed3)III* | |
| OD2641 | | *ltSi884[pOD2010/pSW331; Psas-5::SAS-5(S10A; reencoded); cb-unc-119(+)]II; unc-119(ed3)III* | |
| OD2642 | | *ltSi885[pOD2011/pSW332; Psas-5::SAS-5(S68A; reencoded); cb-unc-119(+)]II; unc-119(ed3)III* | |
| OD2643 | | *ltSi886[pOD2012/pSW333; Psas-5::SAS-5(S71A; reencoded); cb-unc-119(+)]II; unc-119(ed3)III* | |
| OD2647 | | *ltSi890[pOD2019/pSW340; Psas-5::SAS-5(S68A, S71A; reencoded); cb-unc-119(+)]II; unc-119(ed3)III* | |
| OD2644 | | *ltSi887[pOD2013/pSW334; Psas-5::SAS-5(S331A; reencoded); cb-unc-119(+)]II; unc-119(ed3)III* | |
| OD2645 | | *ltSi888[pOD2014/pSW335; Psas-5::SAS-5(S338A; reencoded); cb-unc-119(+)]II; unc-119(ed3)III* | |
| OD2646 | | *ltSi889[pOD2015/pSW336; Psas-5::SAS-5(S340A; reencoded); cb-unc-119(+)]II; unc-119(ed3)III* | |
| OD2536 | | *ltSi357[pY131; Psas-5::SAS-5 reencoded; cb-unc-119(+)]II; unc-119(ed3)III?; ltSi560 [pPLG014; Pmex-5::GFP::his-11::tbb-2_3’UTR, GFP::tbg-1::tbb-2_3’UTR; cb-unc-119(+)]V* | |
| OD2537 | | *ltSi797[pOD1991/pSW311; Psas-5::SAS-5(S10A, S68A, S71A; reencoded); cb-unc-119(+)]II; unc-119(ed3)III?; ltSi560 [pPLG014; Pmex-5::GFP::his-11::tbb-2_3’UTR, GFP::tbg-1::tbb-2_3’UTR; cb-unc-119(+)]V* | |
| OD2538 | | *ltSi802[pOD1996/pSW316; Psas-5::SAS-5(S331A, S338A, S340A; reencoded); cb-unc-119(+)]II; unc-119(ed3)III?; ltSi560 [pPLG014; Pmex-5::GFP::his-11::tbb-2_3’UTR, GFP::tbg-1::tbb-2_3’UTR; cb-unc-119(+)]V* | |
| OD2713 | | *ltSi884[pOD2010/pSW331; Psas-5::SAS-5(S10A; reencoded); cb-unc-119(+)]II; unc-119(ed3)III?; ltSi560 [pPLG014; Pmex-5::GFP::his-11::tbb-2_3’UTR, GFP::tbg-1::tbb-2_3’UTR; cb-unc-119(+)]V* | |
| OD2714 | | *ltSi887[pOD2013/pSW334; Psas-5::SAS-5(S331A; reencoded); cb-unc-119(+)]II; unc-119(ed3)III?; ltSi560 [pPLG014; Pmex-5::GFP::his-11::tbb-2_3’UTR, GFP::tbg-1::tbb-2_3’UTR; cb-unc-119(+)]V* | |
| OD2715 | | *ltSi888[pOD2014/pSW335; Psas-5::SAS-5(S338A; reencoded); cb-unc-119(+)]II; unc-119(ed3)III?; ltSi560 [pPLG014; Pmex-5::GFP::his-11::tbb-2_3’UTR, GFP::tbg-1::tbb-2_3’UTR; cb-unc-119(+)]V* | |
| OD2716 | | *ltSi889[pOD2015/pSW336; Psas-5::SAS-5(S340A; reencoded); cb-unc-119(+)]II; unc-119(ed3)III?; ltSi560 [pPLG014; Pmex-5::GFP::his-11::tbb-2_3’UTR, GFP::tbg-1::tbb-2_3’UTR; cb-unc-119(+)]V* | |
| OD2717 | | *ltSi890[pOD2019/pSW340; Psas-5::SAS-5(S68A, S71A; reencoded); cb-unc-119(+)]II; unc-119(ed3)III?; ltSi560 [pPLG014; Pmex-5::GFP::his-11::tbb-2_3’UTR, GFP::tbg-1::tbb-2_3’UTR; cb-unc-119(+)]V* | |
|  |  | | |
