# Supplementary material for: The kinase ZYG-1 phosphorylates the cartwheel protein SAS-5 to drive centriole assembly in C. elegans
Source: EMBO Rep. 2024 May 14;25(6):2698–721. doi: 10.1038/s44319-024-00157-y (PMC11169420; doi:10.1038/s44319-024-00157-y)
Supplement: Supplementary file 3 — Dataset EV3 [file 44319_2024_157_MOESM3_ESM.docx]

|  | Table EV3 - List of Oligos and crRNAs | | | |
| --- | --- | --- | --- | --- |
|  |  |  | Primers (5' - 3') |  |
|  | Primer Pairs for RFLP analysis for CRISPR/Cas9-mediated Genome editing (5'-3') | | | Restriction Site  (underlined) |
| 1 | For use with gRNAs  numbered 5-13 | FP | GGG AAT GAA TAA TTA CGA CGA CTT ACC CTG CTC | NA |
|  |  | RP | GGG CCG TTT CTT CTT GAA TCT CTG TTC GTA TC |  |
| 2 | For S10A/E | FP | CTG TGT CTC GCT GTC TAT CTG TGT ACA GAG G |  |
|  |  | RP | GGC GAA GCG CTG GAT GCG AAG GTG |  |
| 3 | For S300/304A/E | FP | GCT GAA TGT GAT GCT AAC AGA GCT GCA TAT TC |  |
|  |  | RP | ATC CGG TTC GTA GGT ACG GTA TGA GTC AG |  |
| 4 | For S331/338/340 | FP | ACA GTG GAC AAA TGA GAG ACG TGA TAA TCG TGG |  |
|  |  | RP | CGT TCA GCT GGA GTC TCA GGT ACC GC |  |

|  | Guide RNAs for CRISPR/Cas9-mediated Genome Editing (5'-3') | | |
| --- | --- | --- | --- |
| 5 | S99A | TAACGTTTCCCAAACTGTCG | NA |
| 6 | T101A |  |  |
| 7 | S99/T101A |  |  |
| 8 | T105/S106A | AATTTAGTGGAATTATGCCA |  |
| 9 | T105/S106E |  |  |
| 10 | S99/T101/T105/S106A |  |  |
| 11 | S99/T101/T105/S106E |  |  |
| 12 | SAS-5 83-110 deletion |  |  |
| 13 | SAS-5 65-110 deletion |  |  |
| 14 | S10A/E | TTTTTGAAGTAAATAGAGCA |  |
| 15 | S301/304A/E | CGAAGTATTCGATATGACAA |  |
| 16 | S331/338/340A/E | ATGTCACTCACTCTATCAAA |  |

|  | Repair Templates for Homology Directed Repair for CRISPR/Cas9-mediated Genome Editing (5'-3') (mutations in red) | | |
| --- | --- | --- | --- |
| 17 | S99A | gcatccagcgcttcgccaaaaaacagttgcgtttggaaaaacagtC AAC GTT GCT CAA ACT GTC GAA  gtaattaaattaaaattgttccatggcataattccactaaattatc | HpaI destroyed |
| 18 | T101A | gcatccagcgcttcgccaaaaaacagttgcgtttggaaaaacagtC AAC GTT TCC CAA GCT GTC GAA gtaattaaattaaaattgttccatggcataattccactaaattatc |  |
| 19 | S99/T101A | gcatccagcgcttcgccaaaaaacagttgcgtttggaaaaacagtC AAC GTT GCT CAA GCT GTC GAA gtaattaaattaaaattgttccatggcataattccactaaattatc |  |
| 20 | S99/T101/T105/S106A | ttgtcgtattttgggcagacattgttgaagcaagcactttttt TGA GTT CCG AGC AGC TCC ctgaaagataatttagtggaattatgccatTgaacaattttaa | EcorI Destroyed |
| 21 | S99/T101/T105/S106E | ttgtcgtattttgggcagacattgttgaagcaagcactttttt TGA GTT CCG TTC TTC TCC  ctgaaagataatttagtggaattatgccatTgaacaattttaa |  |
| 22 | SAS-5 83 - 110 deletion | CCGAAACCAAGAAAAGAGCCACCTTCG CAT CCA GCG GTG CTT GCT TCA ACA  ATGTCTGCCCAAAATACGACAA |  |
| 23 | SAS-5 65 - 110 deletion | TTG TCG TAT TTT GGG CAG ACA TTG TTG AAG CAA GCA CGG CTA CTT CTA CAG CGG CGA CAA TTG  GCT GCG TAG ATG GTA CT |  |
| 24 | S10A | tatatcgaaaacaactcaccagttgg ttt ttt Aaa gta aat CGC gca ggg taa gtc gtc gta att att cat tcccgcttaatgc | DraI created |
| 25 | S10E | tatatcgaaaacaactc acc Ggt tggttttttgaagtaaat TTC gcagggtaagtcgtcgtaattattcattcccgcttaatgc | AgeI created |
| 26 | S300/304A | gctttgatgagagaaacacttacggtagaacga TCC atC cgT taC gaT aaT gga ctc gct Gct att gat Gct aga cag  tggacaaatgagagacgtgataatcgtg | XbaI site destroyed |
| 27 | S300/304E | gctttgatgagagaaacacttacggtagaacga TCC atC cgT taC gaT aaT gga ctc gct Gct att gat GAA aga cag  tggacaaatgagagacgtgataatcgtg |  |
| 28 | S331/338/340A | tcataccgtacctacgaaccggatcaaccatgtcac GcT ctc taC caG aag ggC caa GCt att GCT  tattatccgtcagaagcagctggaaaaacg | HaeIII site  created |
| 29 | S331A | tcataccgtacctacgaaccggatcaaccatgtcac GcT ctc taC caG aag ggC caa agt att agc  tattatccgtcagaagcagctggaaaaacg | HaeIII site created |
| 30 | S338A | cgtacctacgaaccggatcaaccatgtcac TcA ctc taC caG aag ggt caa GCt att agc tat tat ccg tca gaa  gcTgctggaaaaacgacagctagaaacaatcgaactggatactac | PvuII site  destroyed |
| 31 | S340A | cgtacctacgaaccggatcaaccatgtcac TcA ctc taC caG aag ggt caa agt att GCT tat tat ccg tca gaa  gcTgctggaaaaacgacagctagaaacaatcgaactggatactac | PvuII site  destroyed |
| 32 | S331/338/340E | tcataccgtacctacgaaccggatcaaccatgtcac GAA ctc taC caG aag ggC caa GAA att GAA  tattatccgtcagaagcagctggaaaaacg | HaeIII site  created |
| 33 | S331E | tcataccgtacctacgaaccggatcaaccatgtcac GAA ctc taC caG aag ggC caa agt att agc  tattatccgtcagaagcagctggaaaaacg | HaeIII site  created |
| 34 | S338E | cgtacctacgaaccggatcaaccatgtcac TcA ctc taC caG aag ggt caa GAA att agc tat tat ccg tca gaa  gcTgctggaaaaacgacagctagaaacaatcgaactggatactac | PvuII site  destroyed |
| 35 | S340E | cgtacctacgaaccggatcaaccatgtcac TcA ctc taC caG aag ggt caa agt att GAA tat tat ccg tca gaa  gcTgctggaaaaacgacagctagaaacaatcgaactggatactac | PvuII site  destroyed |
