# Supplementary figures and images for: The kinase ZYG-1 phosphorylates the cartwheel protein SAS-5 to drive centriole assembly in C. elegans
Source: EMBO Rep. 2024 May 14;25(6):2698–721. doi: 10.1038/s44319-024-00157-y (PMC11169420; doi:10.1038/s44319-024-00157-y)

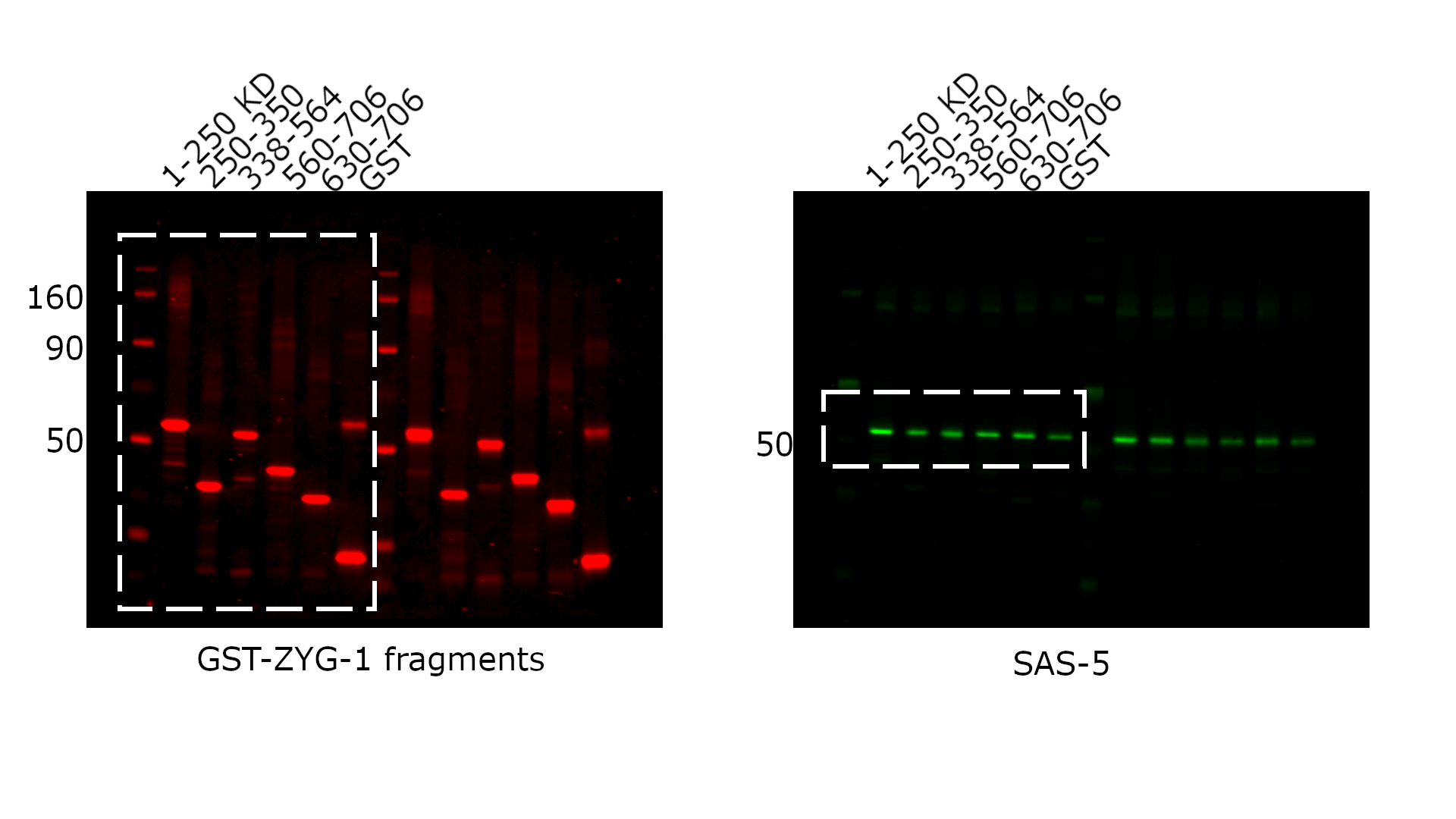

Supplement: Supplementary file 4 — Source data Fig. 1 [file 44319_2024_157_MOESM4_ESM.zip › FIG1/1H/EMBOR-2024-58785_Source data for 1H.tif]

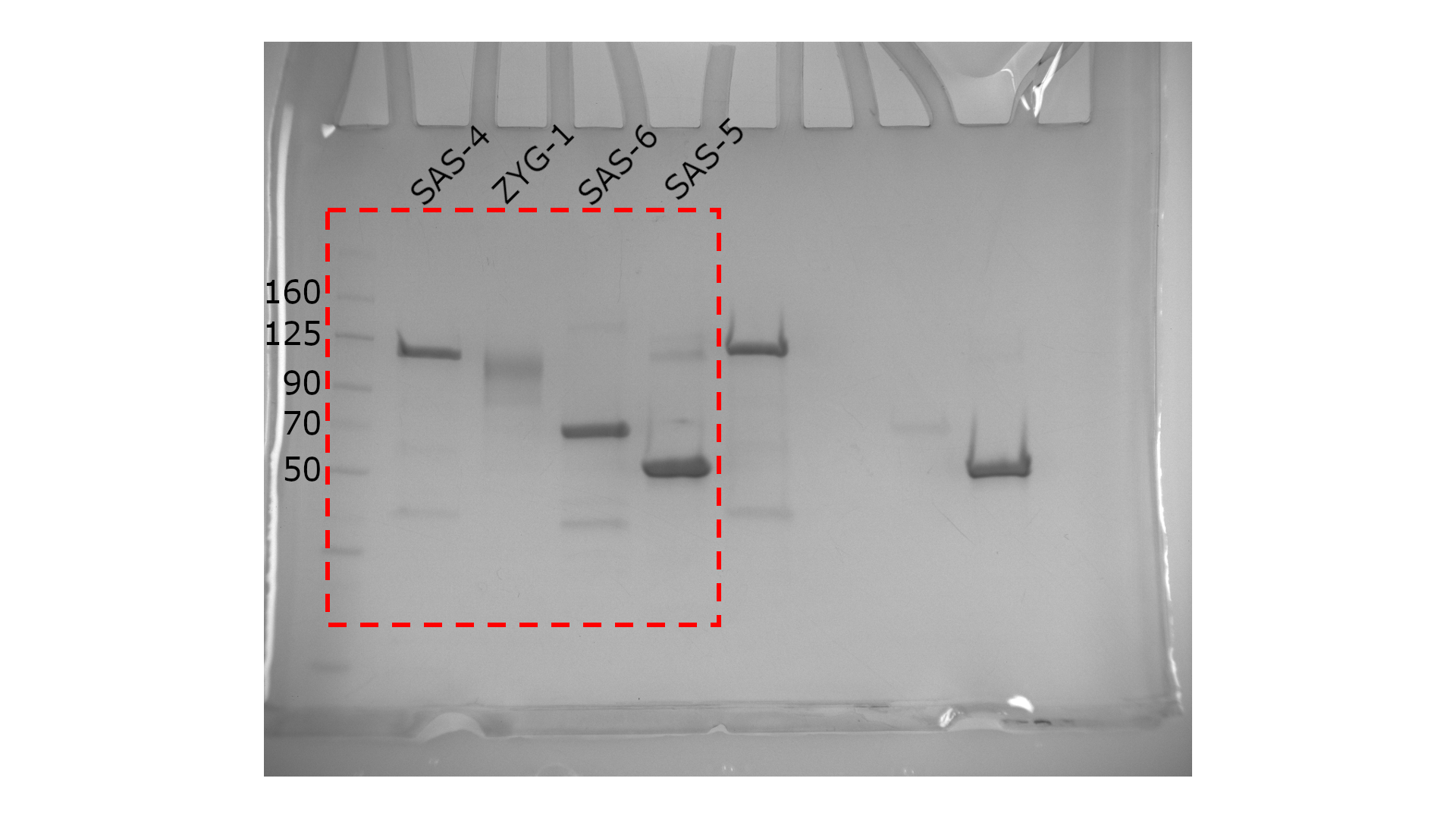

Supplement: Supplementary file 4 — Source data Fig. 1 [file 44319_2024_157_MOESM4_ESM.zip › FIG1/1B/EMBOR-2024-58785_Source data for 1B.tif]

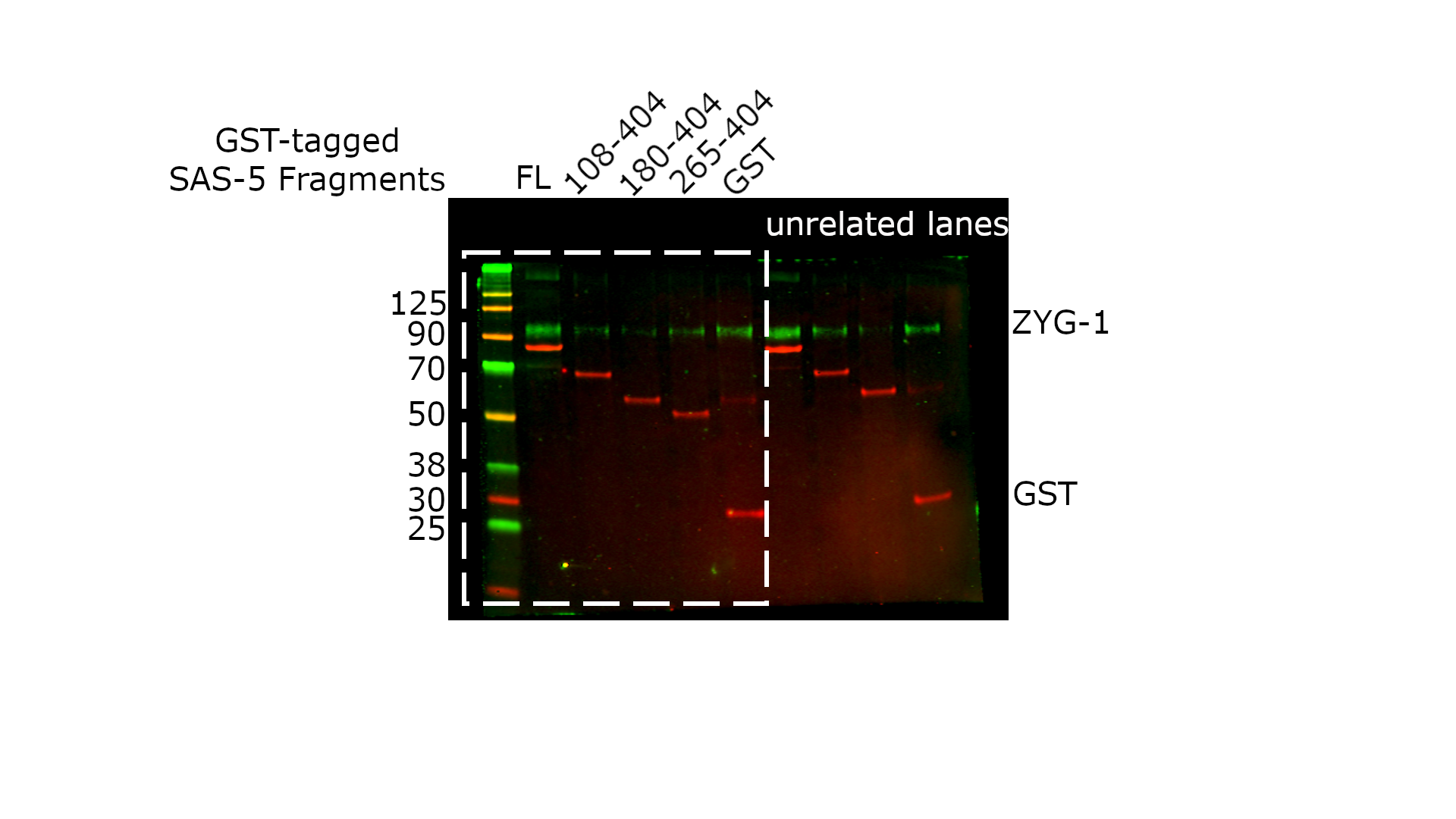

Supplement: Supplementary file 4 — Source data Fig. 1 [file 44319_2024_157_MOESM4_ESM.zip › FIG1/1E/EMBOR-2024-58785_Source data for 1E.tif]

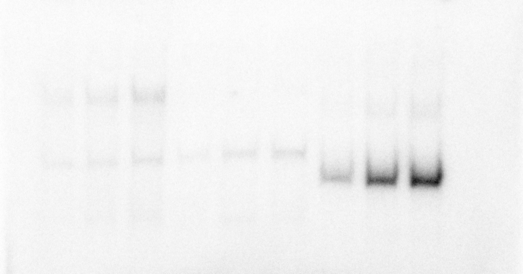

Supplement: Supplementary file 4 — Source data Fig. 1 [file 44319_2024_157_MOESM4_ESM.zip › FIG1/1C/EMBOR-2024-58785_Source data for 1C.tif]

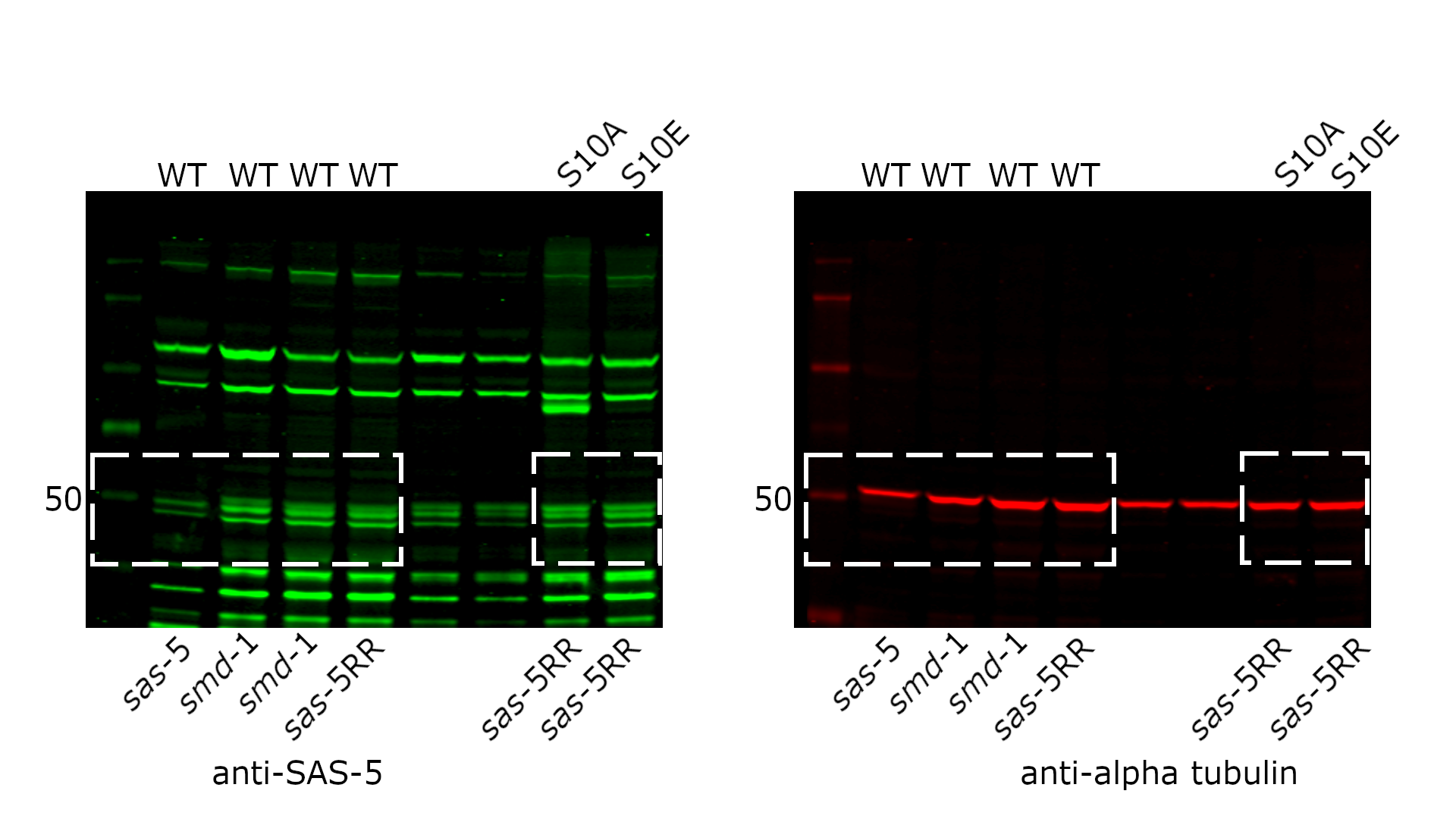

Supplement: Supplementary file 5 — Source data Fig. 2 [file 44319_2024_157_MOESM5_ESM.zip › FIG2/2D/EMBOR-2024-58785_Image Source data for 2D.tif]

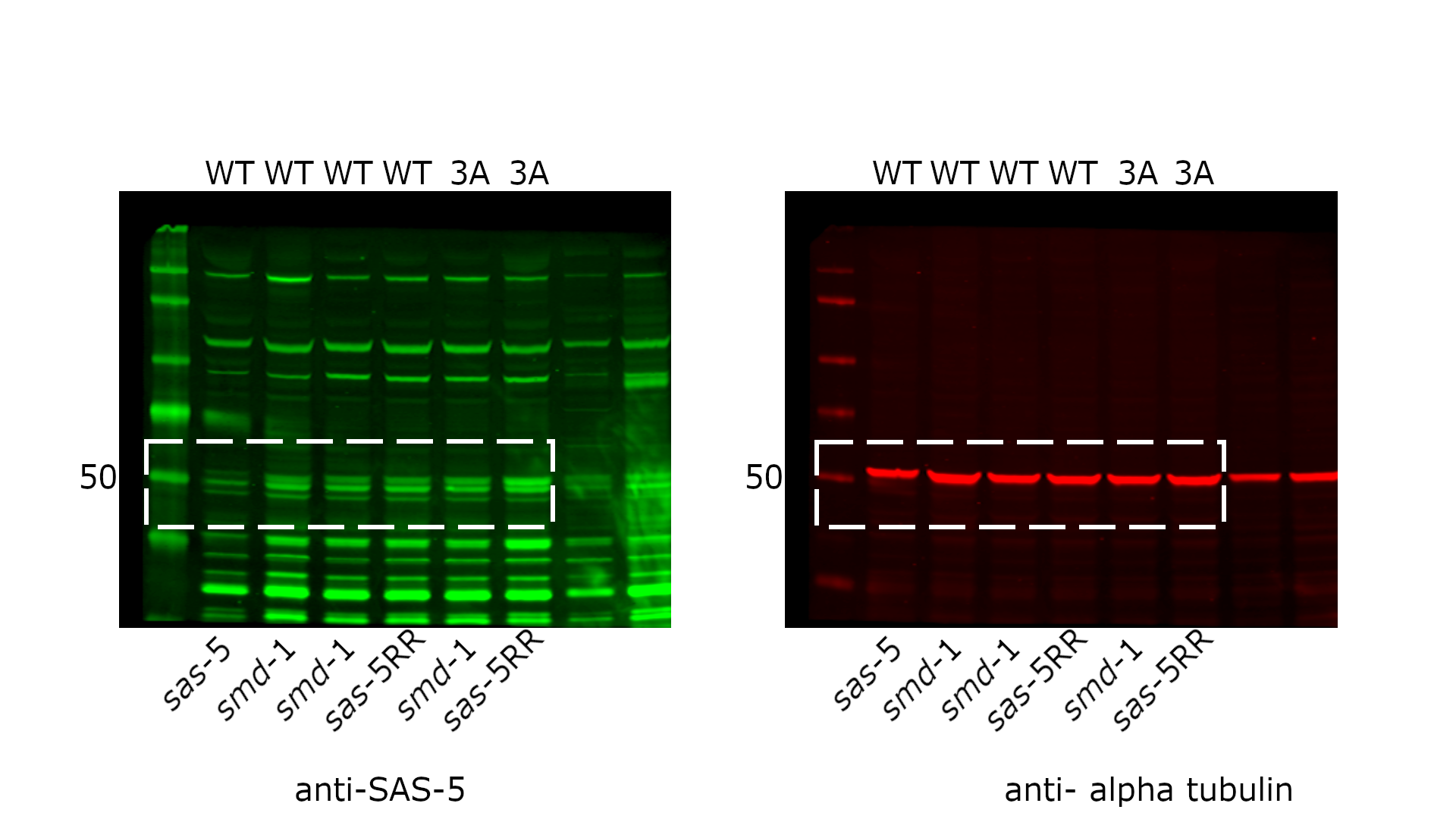

Supplement: Supplementary file 5 — Source data Fig. 2 [file 44319_2024_157_MOESM5_ESM.zip › FIG2/2E/EMBOR-2024-58785_Image Source data for 2E.tif]

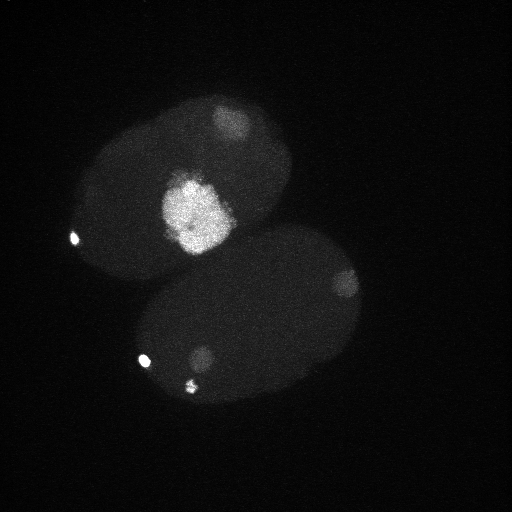

Supplement: Supplementary file 6 — Source data Fig. 3 [file 44319_2024_157_MOESM6_ESM.zip › FIG3/3E/EMBOR-2024-58785_Image Source data for 3E.tif]

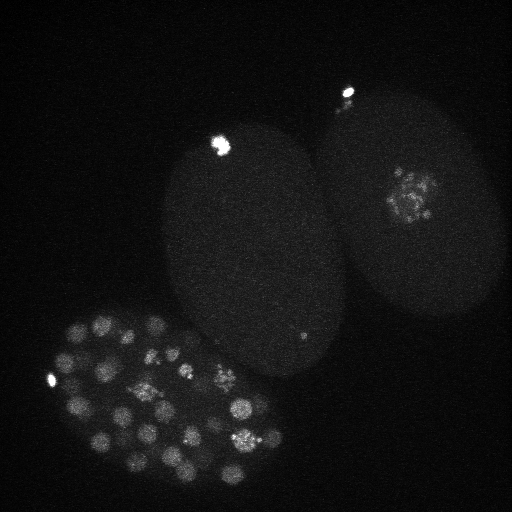

Supplement: Supplementary file 6 — Source data Fig. 3 [file 44319_2024_157_MOESM6_ESM.zip › FIG3/3A/EMBOR-2024-58785_Image Source data for 3A.tif]

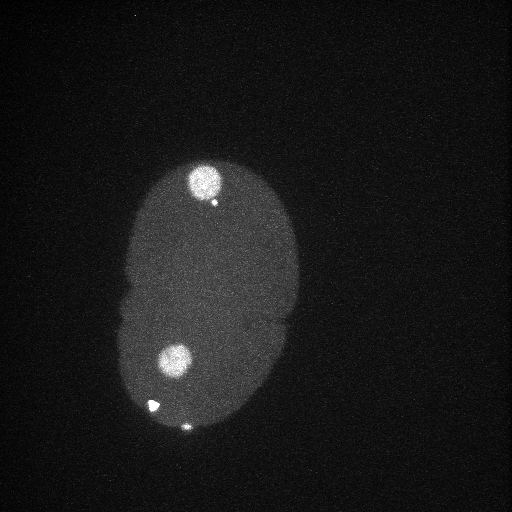

Supplement: Supplementary file 6 — Source data Fig. 3 [file 44319_2024_157_MOESM6_ESM.zip › FIG3/3G/EMBOR-2024-58785_Image Source data for 3G.tif]

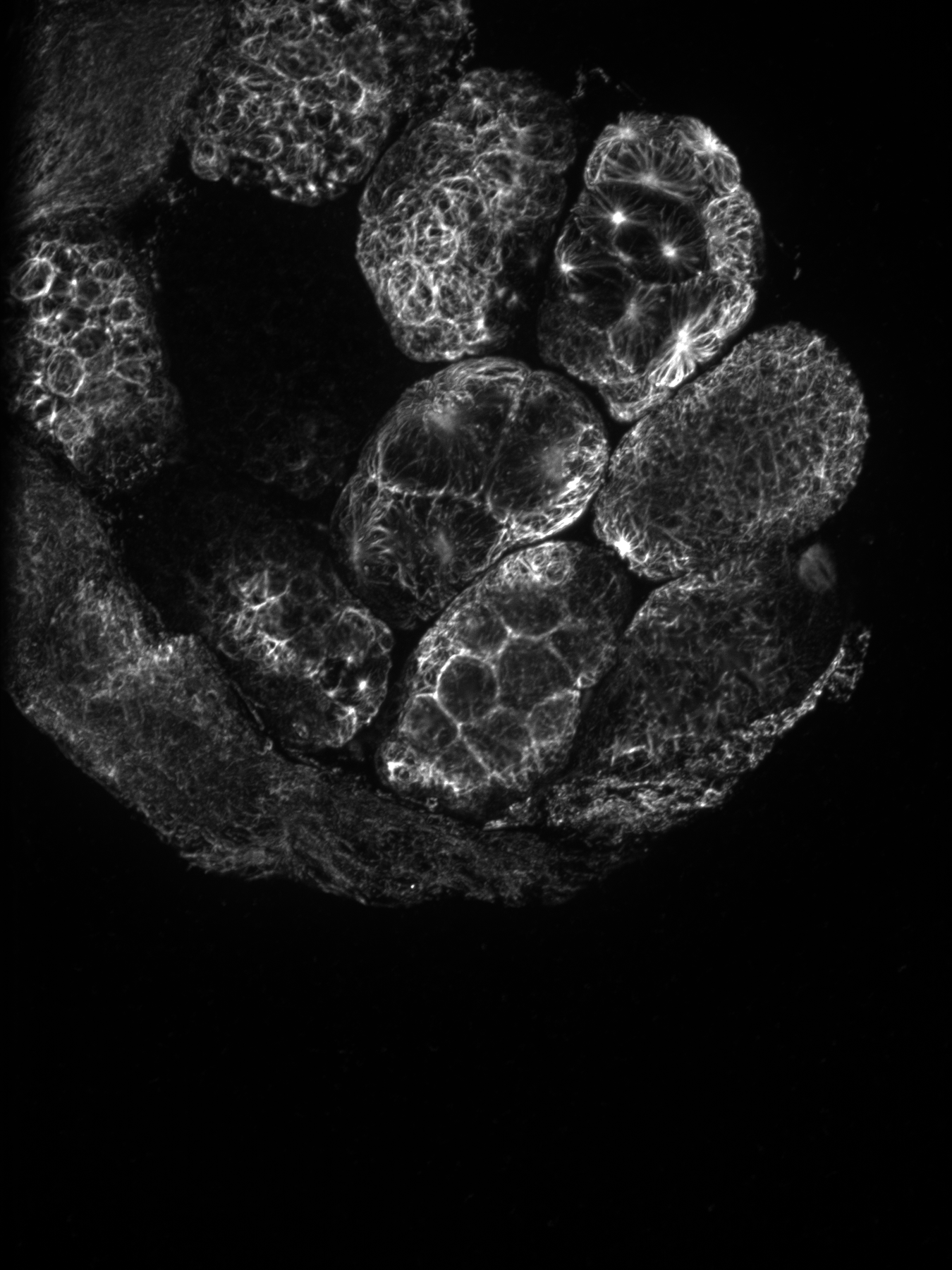

Supplement: Supplementary file 6 — Source data Fig. 3 [file 44319_2024_157_MOESM6_ESM.zip › FIG3/3I (new)/EMBOR-2024-58785_source data Left embryo magenta 3I.tif]

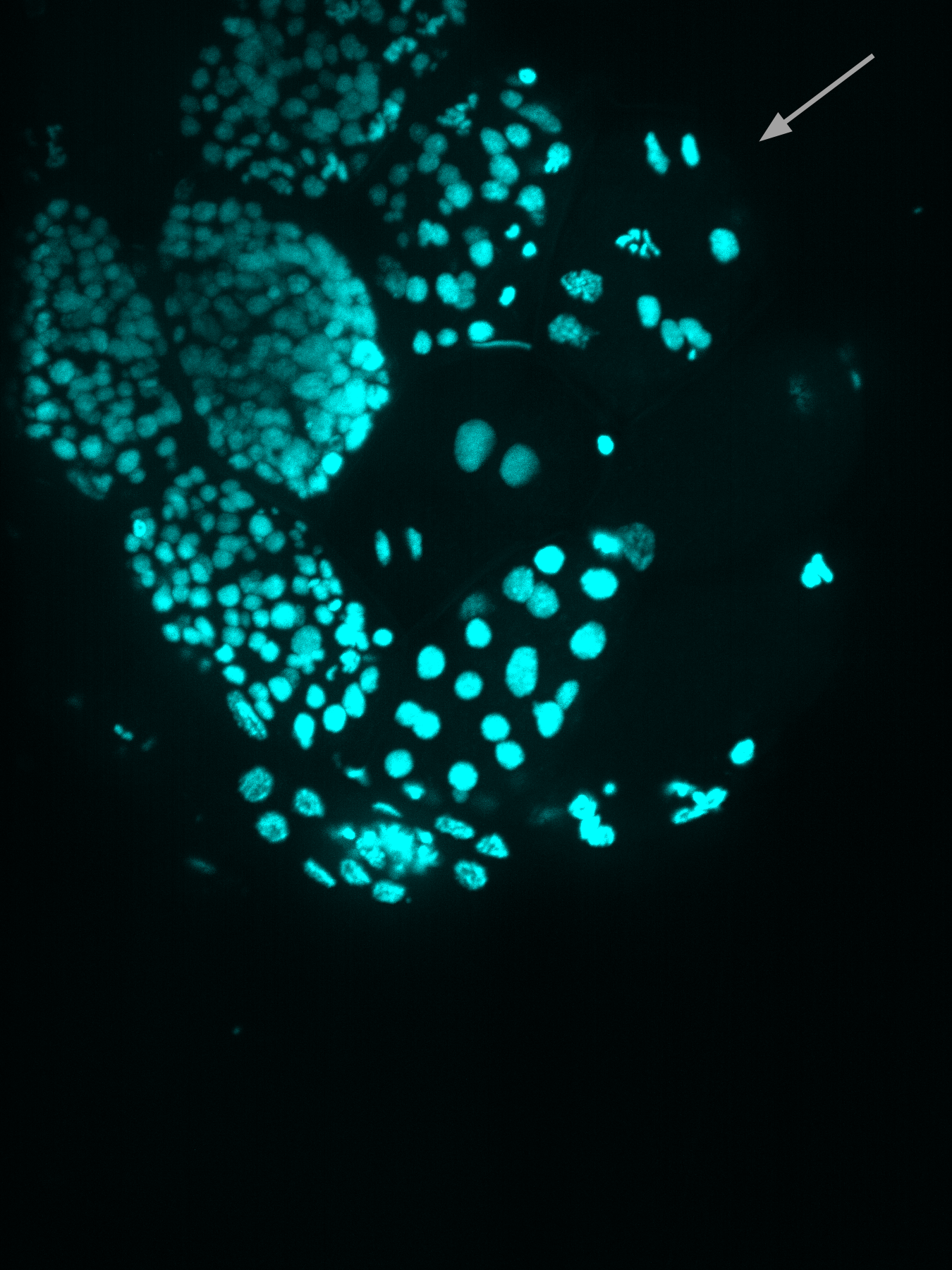

Supplement: Supplementary file 6 — Source data Fig. 3 [file 44319_2024_157_MOESM6_ESM.zip › FIG3/3I (new)/EMBOR-2024-58785_source data left embryo cyan 3I.tif]

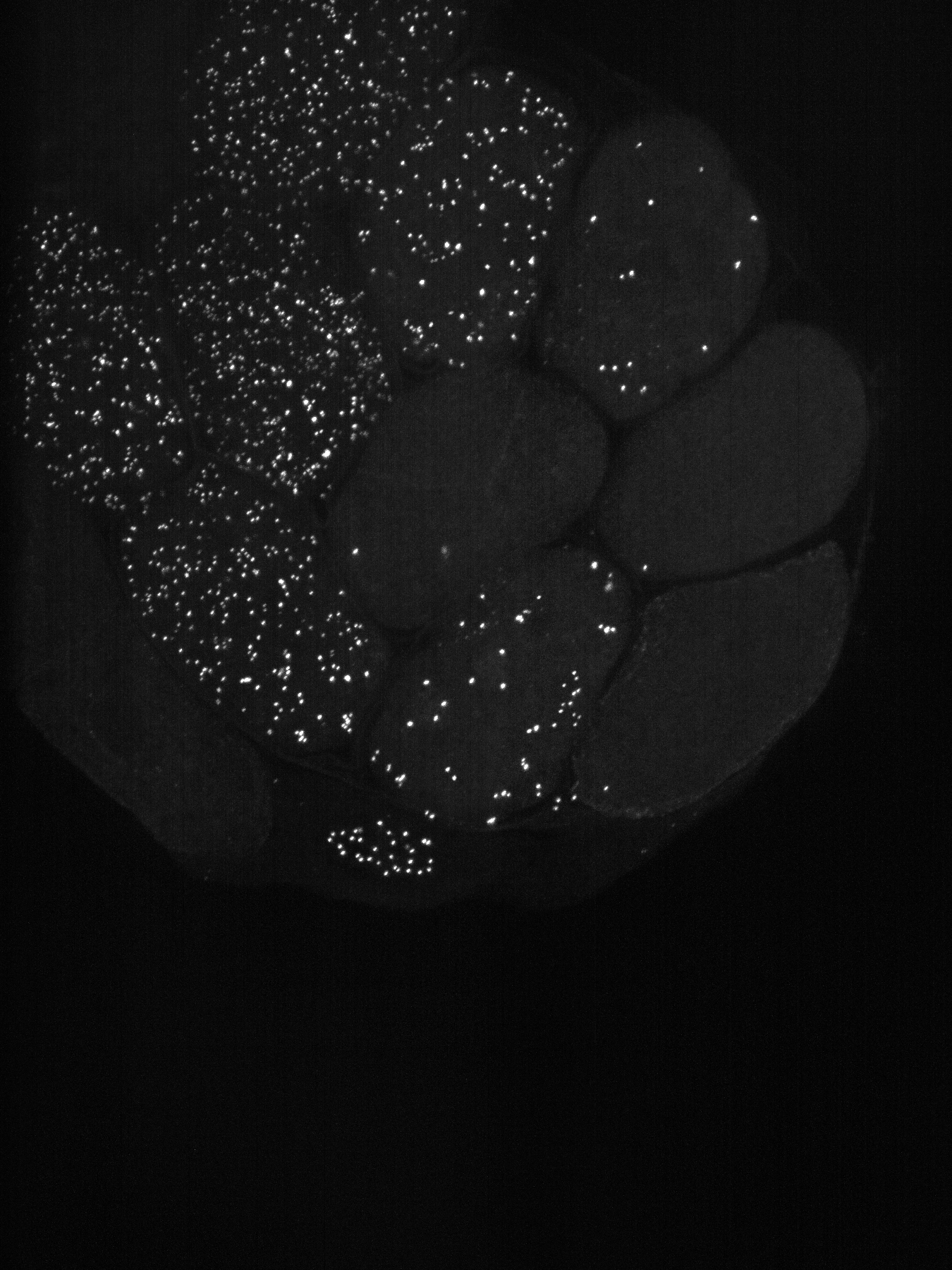

Supplement: Supplementary file 6 — Source data Fig. 3 [file 44319_2024_157_MOESM6_ESM.zip › FIG3/3I (new)/EMBOR-2024-58785_source data Right embryo yellow 3I.tif]

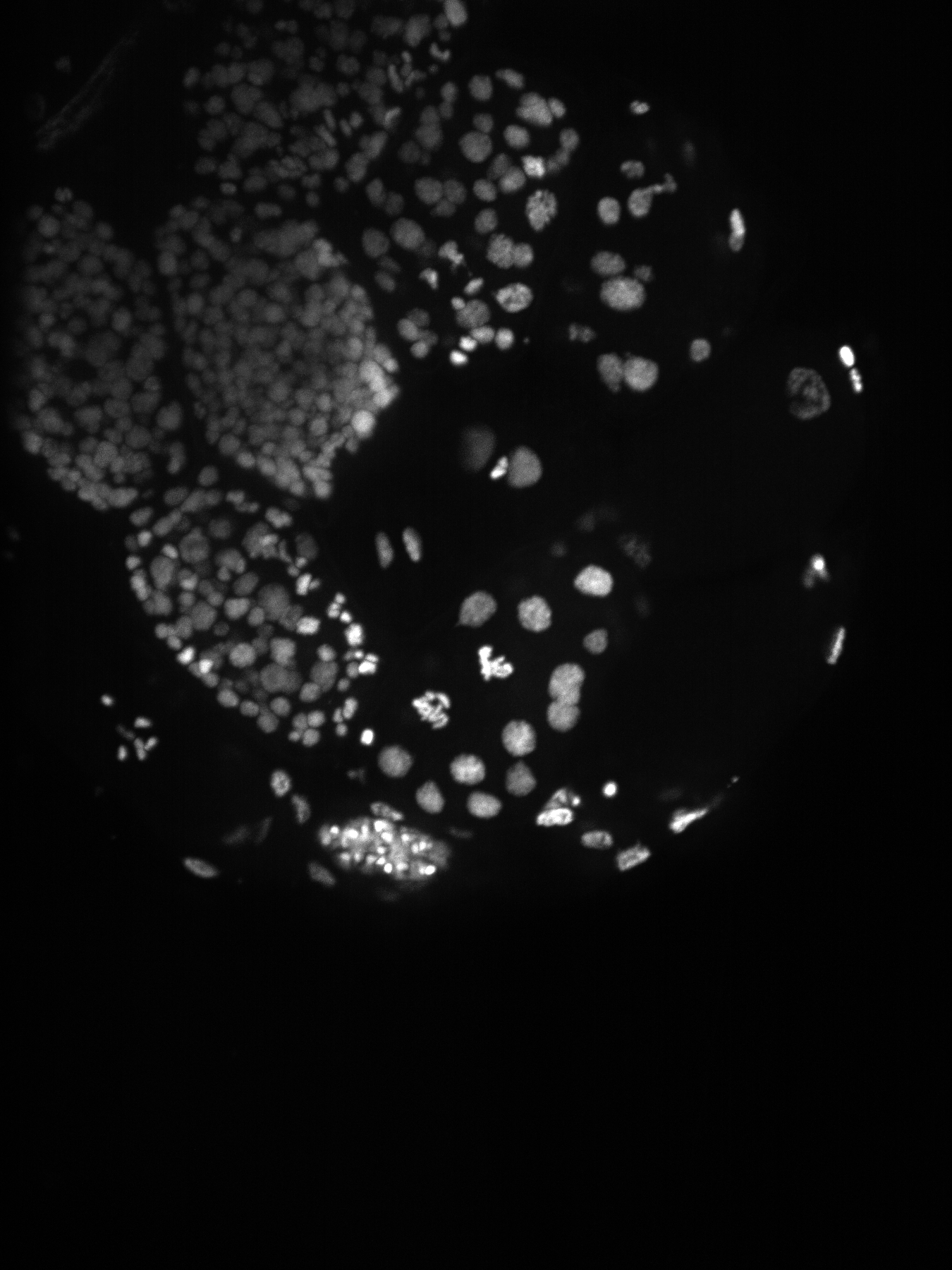

Supplement: Supplementary file 6 — Source data Fig. 3 [file 44319_2024_157_MOESM6_ESM.zip › FIG3/3I (new)/EMBOR-2024-58785_source data Right embryo cyan 3I.tif]

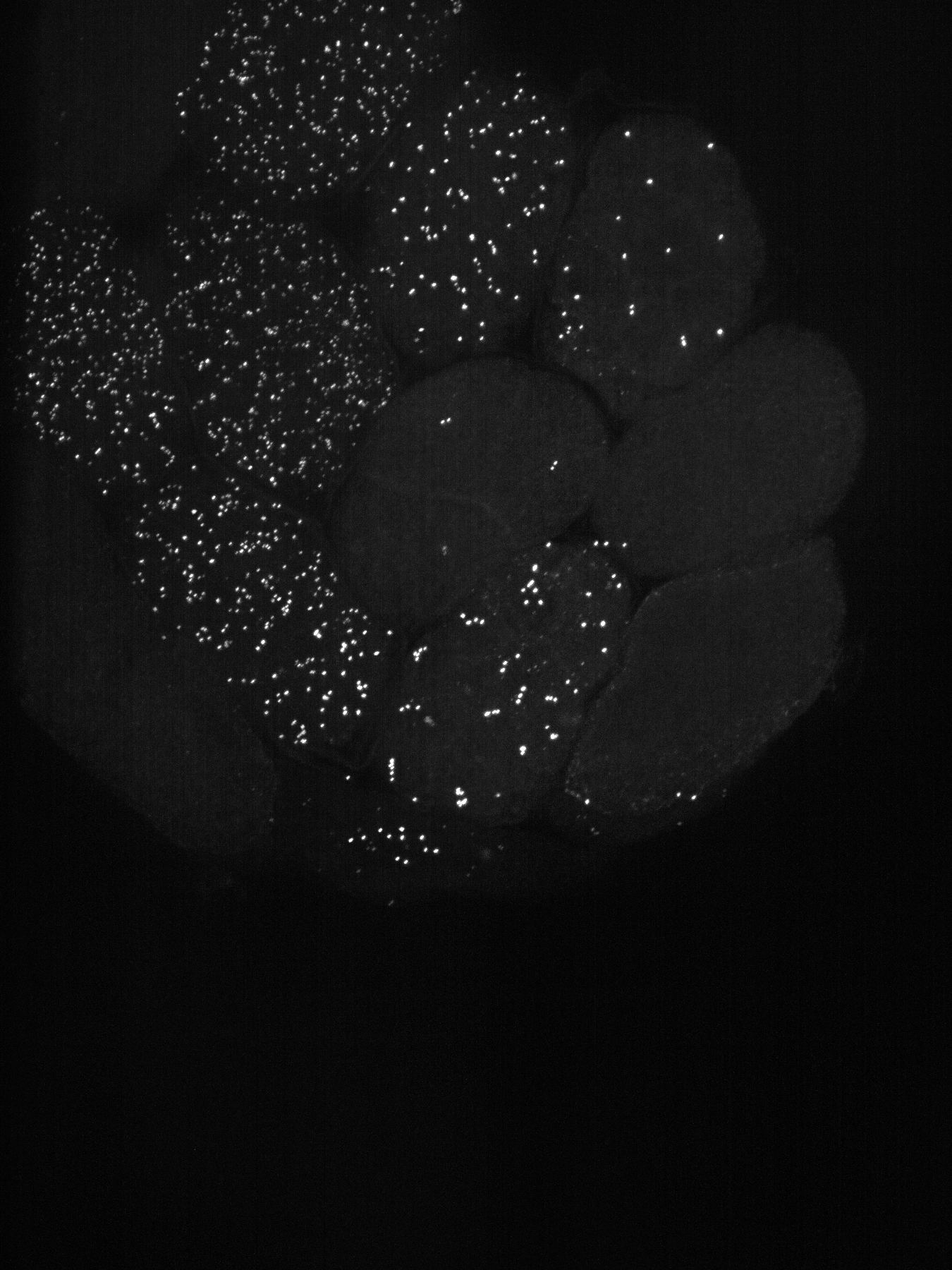

Supplement: Supplementary file 6 — Source data Fig. 3 [file 44319_2024_157_MOESM6_ESM.zip › FIG3/3I (new)/EMBOR-2024-58785_source data Left embryo yellow 3I.tif]

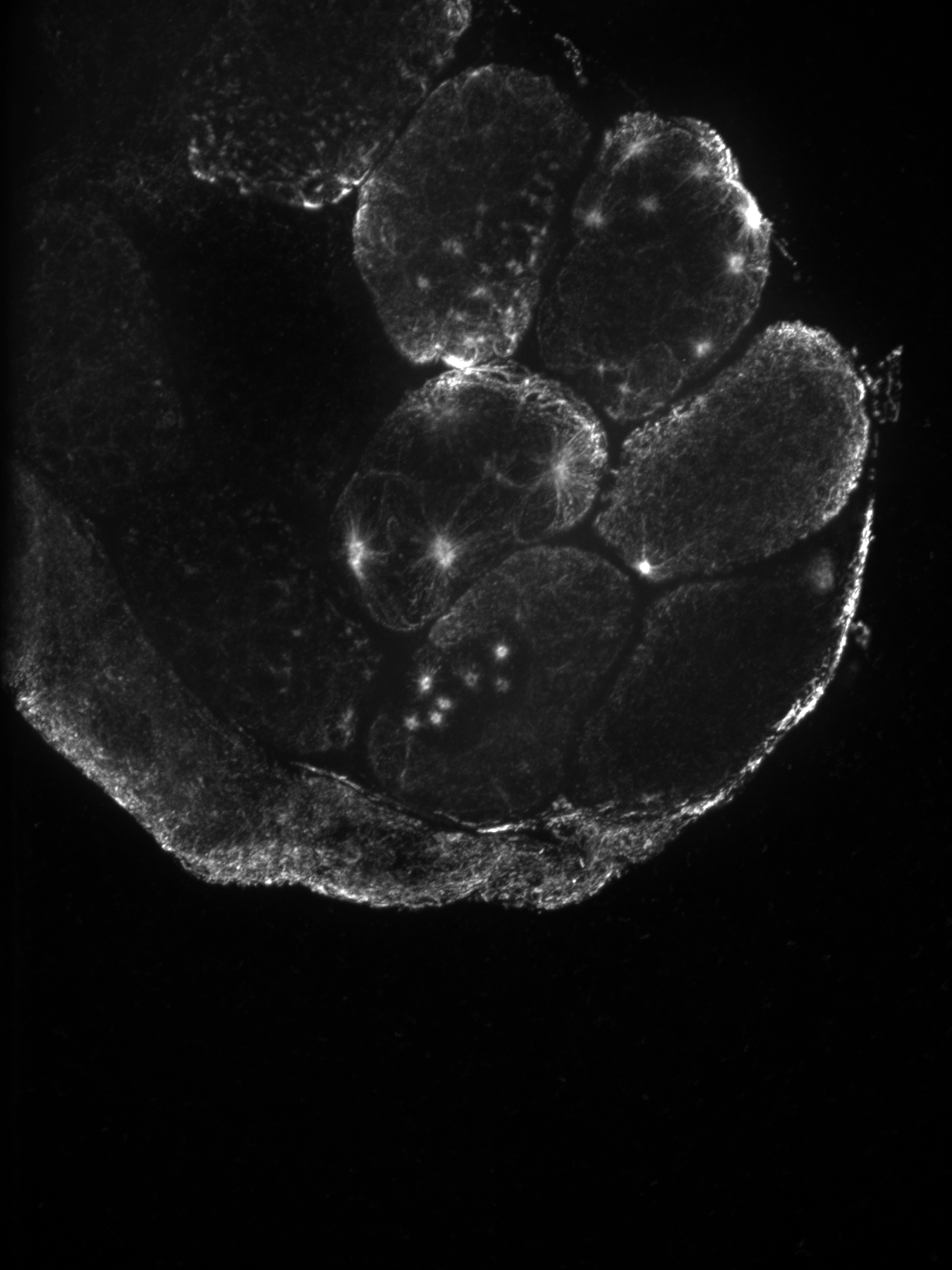

Supplement: Supplementary file 6 — Source data Fig. 3 [file 44319_2024_157_MOESM6_ESM.zip › FIG3/3I (new)/EMBOR-2024-58785_source data Right embryo Magenta 3I.tif]
