# Supplementary material for: The kinase ZYG-1 phosphorylates the cartwheel protein SAS-5 to drive centriole assembly in C. elegans
Source: EMBO Rep. 2024 May 14;25(6):2698–721. doi: 10.1038/s44319-024-00157-y (PMC11169420; doi:10.1038/s44319-024-00157-y)
Supplement: Supplementary file 6 — Source data Fig. 3 [file 44319_2024_157_MOESM6_ESM.zip › FIG3/3C/EMBOR-2024-58785_source data Readme 3C.docx]

This is a maximum intensity projection of planes 10-16

For figure 3C we did the following:

1. We rotated the image to place anterior to left and posterior to right
2. Adjusted brightness and contrast.
3. Cropped.
4. Extracted following frames for figure: 10,24,28, and 34
