# Supplementary material for: The kinase ZYG-1 phosphorylates the cartwheel protein SAS-5 to drive centriole assembly in C. elegans
Source: EMBO Rep. 2024 May 14;25(6):2698–721. doi: 10.1038/s44319-024-00157-y (PMC11169420; doi:10.1038/s44319-024-00157-y)
Supplement: Supplementary file 6 — Source data Fig. 3 [file 44319_2024_157_MOESM6_ESM.zip › FIG3/3A/EMBOR-2024-58785_Image Source data Readme 3A.docx]

Embryo in upper right corner.

This is a maximum intensity projection of planes 3-12

For figure 3A we did the following:

1. rotated the image to place anterior to left and posterior to right
2. Adjusted brightness and contrast.
3. Cropped.
4. Extracted following frames for figure: 5,28, 48, and 63
