# Supplementary material for: The kinase ZYG-1 phosphorylates the cartwheel protein SAS-5 to drive centriole assembly in C. elegans
Source: EMBO Rep. 2024 May 14;25(6):2698–721. doi: 10.1038/s44319-024-00157-y (PMC11169420; doi:10.1038/s44319-024-00157-y)
Supplement: Supplementary file 6 — Source data Fig. 3 [file 44319_2024_157_MOESM6_ESM.zip › FIG3/3I (new)/EMBOR-2024-58785_source dataRead Me 3I.docx]

For SPOT::SAS-6 stained embryos:

1. Split colors.
2. Merge CMYK: DNA Cyan, SPOT::SAS-6 yellow, microtubules magenta.
3. Max intensity projection 1-27 (left embryo) or 33-55 (right embryo).
4. Adjust Brightness and Contrast.
5. Rotate -103°.
6. Crop to 475 x 300 pixels.
7. Enlarged 120% within Gimp.
