# Supplementary material for: The kinase ZYG-1 phosphorylates the cartwheel protein SAS-5 to drive centriole assembly in C. elegans
Source: EMBO Rep. 2024 May 14;25(6):2698–721. doi: 10.1038/s44319-024-00157-y (PMC11169420; doi:10.1038/s44319-024-00157-y)
Supplement: Supplementary file 7 — Source data Fig. 4 [file 44319_2024_157_MOESM7_ESM.zip › FIG4/4B/EMBOR-2024-58785_source data README for Fig 4B.docx]

The embryo at the top right of the field

1. Maximum intensity projection of focal planes 2 through 8.
2. Rotated to bring the anterior with polar body to left hand side.
3. Cropped.
4. Adjusted brightness and contrast.
5. Extracted following frames for figure: 1, 5, 7 and 9.
