# Supplementary material for: The kinase ZYG-1 phosphorylates the cartwheel protein SAS-5 to drive centriole assembly in C. elegans
Source: EMBO Rep. 2024 May 14;25(6):2698–721. doi: 10.1038/s44319-024-00157-y (PMC11169420; doi:10.1038/s44319-024-00157-y)
Supplement: Supplementary file 7 — Source data Fig. 4 [file 44319_2024_157_MOESM7_ESM.zip › FIG4/4C/EMBOR-2024-58785_source data README for Fig 4C.docx]

The embryo at the center of the field

1. The file was converted to stack from hyperstack.
2. The file reconverted to stack, no. of Z slice – 18, no. of images – 20.
3. Arrange channels to assign green to red and red to green.
4. Split channels.
5. Maximum intensity projection of focal planes 2 through 8.
6. Cropped.
7. Extracted following frames for figure: 1, 3, 4, and 5.
