# Supplementary material for: The kinase ZYG-1 phosphorylates the cartwheel protein SAS-5 to drive centriole assembly in C. elegans
Source: EMBO Rep. 2024 May 14;25(6):2698–721. doi: 10.1038/s44319-024-00157-y (PMC11169420; doi:10.1038/s44319-024-00157-y)
Supplement: Supplementary file 8 — Source data Fig. 5 [file 44319_2024_157_MOESM8_ESM.zip › FIG5/5B/EMBOR-2024-58785_source data README for Fig 5B.docx]

The embryo at the center of the field

1. Maximum intensity projection of focal planes 9 through 15.
2. Rotated to bring the anterior with polar body to left hand side.
3. Cropped.
4. Adjusted brightness and contrast.
5. Extracted following frames for figure: 4, 8, 11, and 13.
