# Supplementary material for: The kinase ZYG-1 phosphorylates the cartwheel protein SAS-5 to drive centriole assembly in C. elegans
Source: EMBO Rep. 2024 May 14;25(6):2698–721. doi: 10.1038/s44319-024-00157-y (PMC11169420; doi:10.1038/s44319-024-00157-y)
Supplement: Supplementary file 10 — Expanded View Figures [file 44319_2024_157_MOESM10_ESM.pdf]

Expanded View Figures

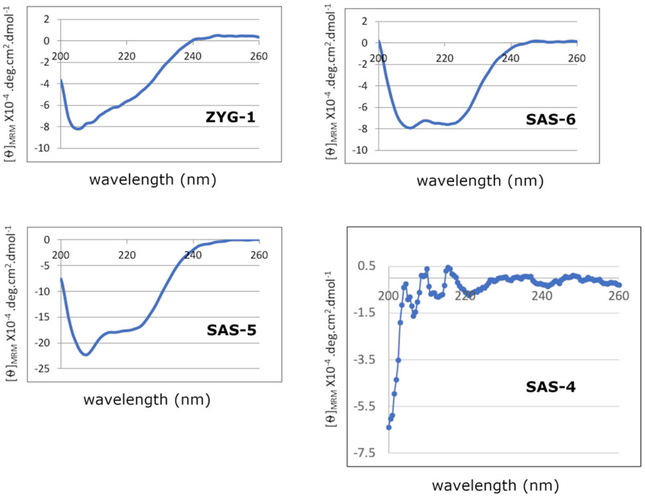

Figure EV1. Purification and characterization of recombinant core centriolar proteins.

Circular dichroism spectra of the refolded proteins indicate secondary structural elements are present in the refolded proteins. The spectrum of ZYG-1 indicates that the protein consists of both alpha-helices and beta-sheets, while the spectra of SAS-5 and SAS-6 indicate that the proteins consist predominantly of alpha-helices (dual peak minima at wavelengths around 208 and 222 nm). SAS-4 appears to lack secondary structural elements.

 © This is a U.S. Government work and not under copyright protection in the US; foreign copyright protection may apply

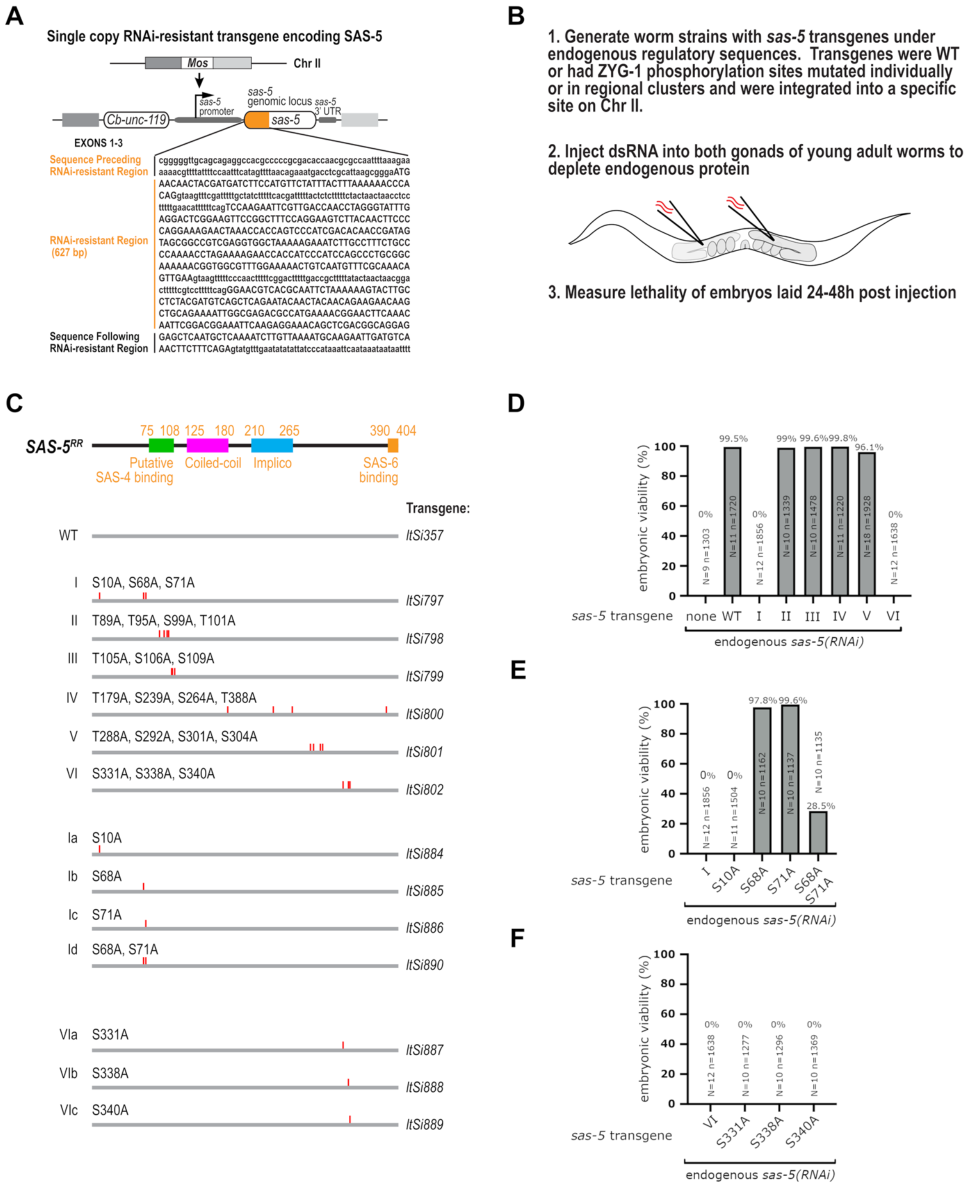

Figure EV2. Screen of phospho-acceptor sites using mutant transgenes.

(**A**) Schematic of the *sas-5* transgene integrated on chromosome II. The recoded portion of the transgene is indicated and allowed us to use RNAi to specifically target either the endogenous gene or the transgene itself. (**B**) Scheme used to assay embryonic lethality of control and mutant transgenes. (**C**) The initial screen utilized 6 mutant transgenes (I–VI), each comprising multiple serine-to-alanine mutations. The identity of the residues targeted in each construct is shown. (**D–F**) Embryonic viability of strains carrying the indicated versions of the *sas-5* transgene and subjected to *sas-5(RNAi)*. *N*=number of hermaphrodites and *n* = number of embryos counted. Note that the only single mutants that result in a complete loss of viability are S10A, S331A, S338A, and S340A.

© This is a U.S. Government work and not under copyright protection in the US; foreign copyright protection may apply

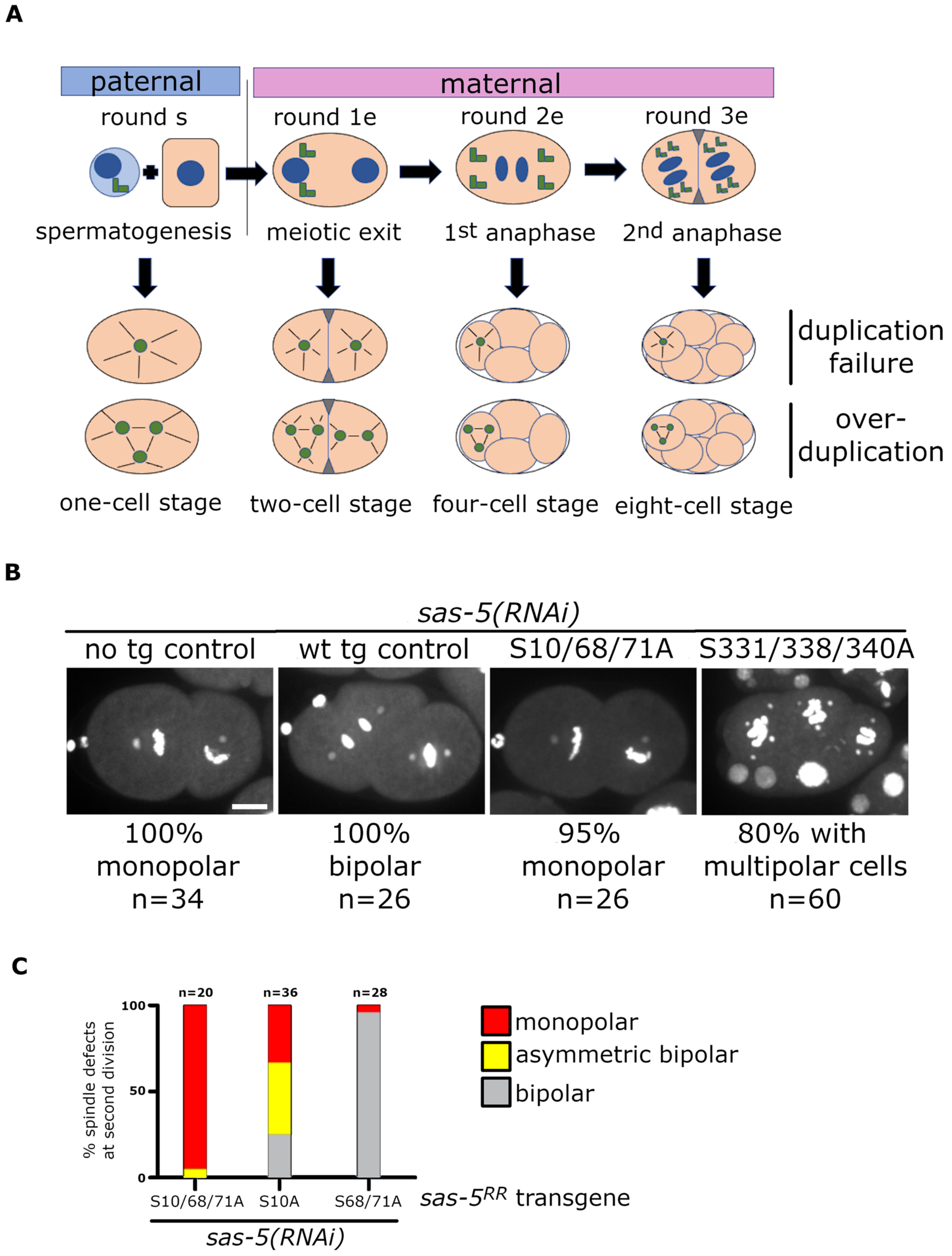

 © This is a U.S. Government work and not under copyright protection in the US; foreign copyright protection may apply

Figure EV3. Centriole duplication errors in strains expressing mutant sas-5 transgenes.

(**A**) An explanation of how errors in centriole duplication (duplication failure or overduplication) manifest as spindle assembly defects. The effect of these errors on spindle assembly are not immediately apparent as the mother and daughter centrioles remain in close association until the ensuing cell cycle when they separate in preparation for the next round of spindle assembly. This has special consequences for the early embryo where the first pair of centrioles are inherited exclusively from the sperm, while the components required for duplication are provided maternally. Hence paternal defects that occur during spermatogenesis (round s) lead too few or too many sperm centrioles and ultimately mono- and multipolar spindles in the one-cell embryo. In contrast maternal defects in centriole assembly which can occur during the first (e1), second (e2), or third round (e3 and so forth), result in spindle defects during the two-, four-, and eight-cell stages respectively. Depending on the severity of the defect, mono- or multipolar spindles might be observed in some or all of the cells of the embryo. For simplicity, spindle defects are only depicted in one of the cells of four- and eight-cell stage embryos. (**B**) Spindle assembly defects in strains expressing wild-type and mutant versions of the recoded *sas-5* transgene. All strains were subject to *sas-5(RNAi)* targeting the endogenous gene. The no transgene control possesses all monopolar spindles at the two-cell stage due a complete block in the first (1e) round of centriole assembly. In contrast the strain with a wild-type copy of the transgene only possesses bipolar spindles demonstrating the ability of the transgene to escape RNAi-based silencing. The strain expressing the S10/68/71A triple mutant exhibits a nearly complete block in centriole assembly leading to mostly monopolar spindles at the two-cell stage. The strain expressing the S331/338/340 A triple mutant exhibits a high frequency of multipolar spindles indicating overduplication occurred during the early embryonic divisions. The S331A, S338A and S340A single mutants all exhibited a low frequency of multipolar spindles. Bar, 10 µm. (**C**) Quantification of spindle defects in S10/68/71A, S10A, and S68/71A expressing strains. Asymmetric spindles contain one normal sized pole and a second much smaller pole. Such a defect has been attributed to a partial block in centriole assembly.

© This is a U.S. Government work and not under copyright protection in the US; foreign copyright protection may apply

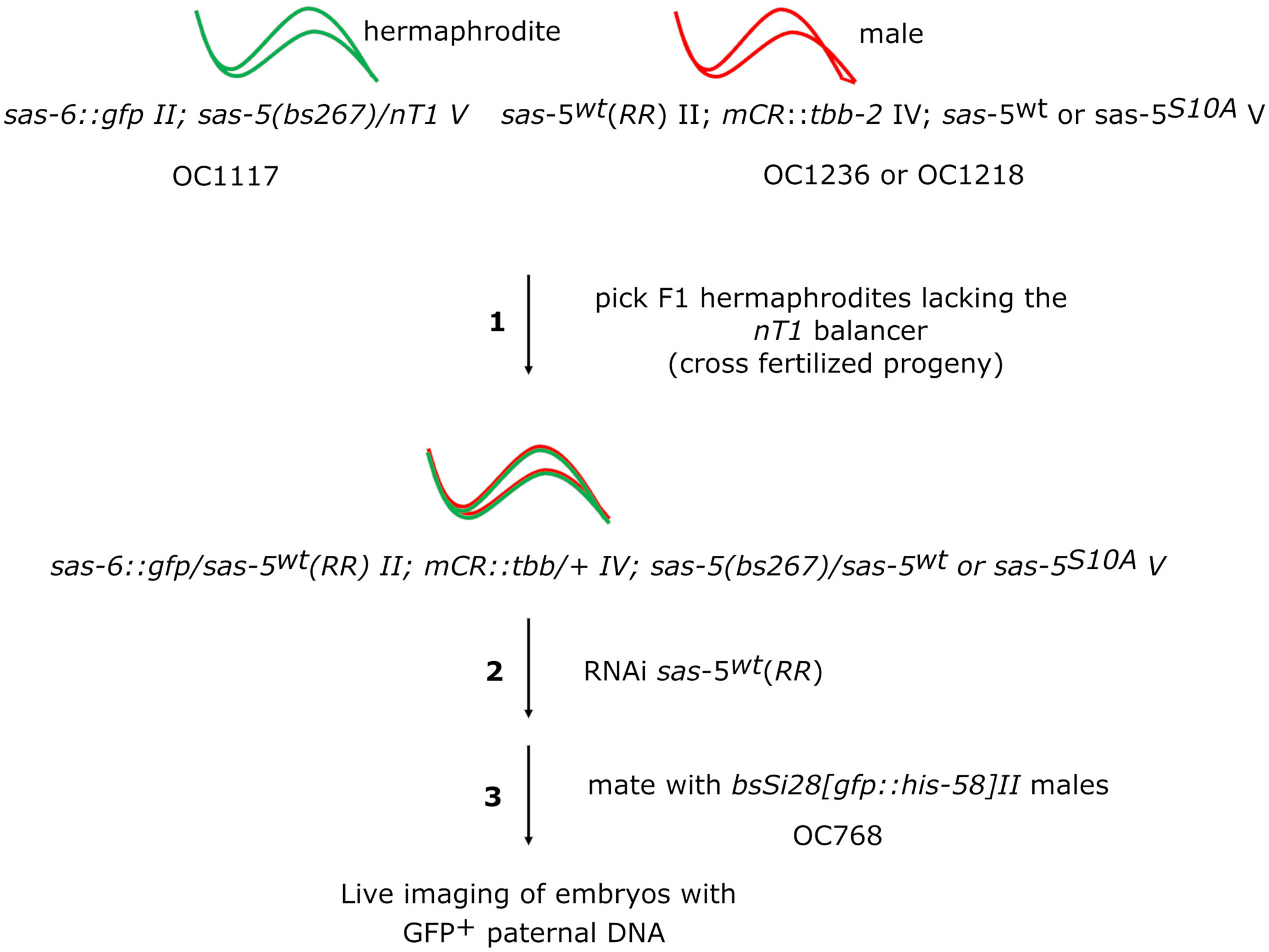

Figure EV4. Genetic Scheme for SAS-6 recruitment assay.

The SAS-6 recruitment assay was performed with hermaphrodites carrying both the *sas-6::gfp* and rescuing recoded *sas-5^{WT}(RR)* transgenes (red and green worm). Because these are both integrated at the same position on chromosome II we had to use following strategy to construct the desired hermaphrodites. Hermaphrodites of strain OC1117, carrying the *sas-6::gfp* transgene and heterozygous for the *sas-5* null allele *bs267* were crossed to males carrying the *sas-5^{wt}(RR)* rescuing transgene and an mCherry (mCR) ::tubulin transgene (1). These males were either wild-type at the endogenous *sas-5* locus (strain OC1236) or carried the *sas-5^{S10A}* mutation (strain OC1218). The resulting hermaphrodite cross progeny were treated with RNAi targeting the *sas-5^{wt}(RR)* transgene (2) and mated to males of strain OC768 which expressed GFP::histone (3). Zygotes expressing GFP positive paternal chromatin were imaged.

 © This is a U.S. Government work and not under copyright protection in the US; foreign copyright protection may apply
